# Comment on: “Effect of melatonin on postoperative cognitive function in elderly patients submitted to transurethral resection of the prostate under spinal anesthesia”

**DOI:** 10.1016/j.clinsp.2026.100954

**Published:** 2026-04-24

**Authors:** Isabelly Victoria Simões Melchiori, Marcelo Augusto Magalhães de Arruda

**Affiliations:** Hospital das Clínicas da Faculdade de Medicina da Universidade de São Paulo (HCFMUSP), São Paulo, SP, Brazil

We read with interest the randomized, double-blind trial by Tavares et al. evaluating perioperative melatonin for elderly men undergoing TURP under spinal anesthesia, with neuropsychological assessment preoperatively and at 21- and 180-days postoperatively. The study found no difference in delayed neurocognitive recovery at 21-days, no cases of longer-term PND at 180-days, and small domain-specific advantages (delayed-recall FOME and Digit Span) in the melatonin group, alongside an unexpected global cognitive improvement at 180-days in both groups.^1^

The trial addresses a clinically relevant and mechanistically plausible hypothesis: hospitalization/sleep disruption as a modifiable contributor to perioperative brain vulnerability, with melatonin as a low-cost intervention. This aligns with contemporary perioperative brain health frameworks that emphasize screening and addressing modifiable risk factors (including sleep) rather than focusing only on anesthetic exposure.^2^^,^^3^ Restricting the cohort to a single, common procedure (TURP) and regional anesthesia reduces heterogeneity from anesthetic technique and major pain/bleeding ‒ important given how multifactorial PND is.^4^ Also, authors used a relatively comprehensive cognitive battery and attempted to mitigate learning effects via alternative test versions, improving interpretability compared with studies relying only on global screening tools.^1^^,^^4^

A few methodological aspects limit the strength of inference of the present trial and merit discussion in light of recent literature. First, the study did not reach its planned sample size (≈426 participants) and was terminated early after a futility analysis with substantially fewer enrolled patients. Although futility analyses are methodologically acceptable, early stopping combined with low event rates substantially increases the risk of type II error. This is particularly relevant in the context of melatonin research, where contemporary meta-analyses consistently suggest small-to-moderate effect sizes on perioperative neurocognitive outcomes, with estimates highly sensitive to baseline risk, outcome definitions, and concomitant interventions. Consequently, the absence of a statistically significant global effect in an underpowered study should be interpreted cautiously rather than as definitive evidence of inefficacy.^5^^,^^6^

Second, outcome definition and timing remain challenging in a field undergoing conceptual and methodological evolution. The use of a 21-day endpoint for delayed neurocognitive recovery and a 180-day endpoint for longer-term impairment is reasonable; however, comparability across studies is limited by heterogeneity in cognitive batteries, diagnostic thresholds, and follow-up intervals. Recent reviews emphasize that such variability is a major contributor to discordant findings and may obscure domain-specific cognitive trajectories. Within this framework, the isolated improvements observed in attention and memory domains in the melatonin group may be clinically relevant, yet they require cautious interpretation given issues of multiplicity and limited statistical power.^4^

A particularly relevant aspect of this cohort is the high prevalence of baseline cognitive impairment. This raises the possibility that postoperative cognitive trajectories reflect regression to the mean, learning effects from repeated testing, or improvement in chronic contributors to cognitive dysfunction ‒ most notably sleep fragmentation associated with lower urinary tract symptoms and nocturia ‒ rather than a direct effect of surgery, anesthesia, or the intervention itself. The authors’ hypothesis that symptom relief after TURP may improve sleep and, secondarily, cognitive performance is biologically plausible and supported by contemporary LUTS literature linking sleep disturbance to symptom burden and broader cerebrovascular and neurodegenerative risk pathways. Importantly, if the surgical intervention itself improves sleep and quality of life, any incremental benefit of melatonin may be attenuated unless targeted to patients with persistent postoperative sleep disruption or heightened circadian vulnerability. This interpretation is consistent with delirium-prevention literature, in which multimodal sleep-promoting strategies outperform isolated pharmacologic interventions in unselected populations.^7^, ^8^, ^9^, ^10^

Furthermore, the interpretation of the 180-day findings is further complicated by non-trivial missing data. Assumptions regarding missing-at-random mechanisms and the choice of imputation strategy can materially influence longitudinal cognitive estimates, particularly when an across-the-board cognitive improvement is observed. A more explicit sensitivity analysis ‒ such as alternative multiple imputation models or pattern-mixture approaches ‒ would strengthen confidence in the conclusion that global cognitive improvement occurred independently of group allocation.^1^

From a perioperative care standpoint, the study may be most impactful not for the negative melatonin result, but for highlighting i) The burden of baseline cognitive vulnerability in men presenting for TURP, and ii) The possibility that improving a chronic sleep-disrupting condition (LUTS/nocturia) can shift cognitive performance over months. This fits current calls to operationalize perioperative brain health through preoperative cognitive screening, delirium-risk assessment, and optimization of sleep and other modifiable factors.^2^^,^^3^ In other words, the trial supports a reframing: “brain outcomes” after TURP may be strongly influenced by the underlying urologic/sleep phenotype, suggesting that future neuroprotection trials should stratify by baseline sleep/LUTS severity and incorporate objective or validated sleep measures (e.g., actigraphy, PROMIS sleep scales) alongside cognitive endpoints.^7^^,^^10^

Future trials could increase yield by 1) Enriching for high-risk patients (severe sleep disturbance, preoperative cognitive impairment, prior delirium, frailty), 2) Embedding standardized delirium assessment early postoperatively (where sleep/circadian interventions often show the clearest signal), and 3) Testing dosing/timing regimens that better match circadian biology (chronotype-informed timing; longer postoperative courses), ideally within bundled sleep-promoting protocols rather than melatonin alone.^5^^,^^6^^,^^10^

In conclusion, Tavares et al. provide a well-conceived, procedure-focused randomized trial that ‒ despite limitations from early stopping and complex baseline vulnerability ‒ advances an important clinical message: perioperative cognitive trajectories may be strongly coupled to sleep and LUTS phenotypes, and “brain health” initiatives should integrate these factors into both routine care and future intervention studies.^1^, ^2^, ^3^^,^^7^^,^^10^

## Authors’ contributions

Isabelly Victoria Simões Melchiori: Conceptualization; Writing-original draft; Writing-review & editing.

Marcelo Augusto Magalhães de Arruda: Conceptualization; Writing-review & editing.

## Funding

This study received no external funding.

## Data availability

All data discussed are derived from the original article cited in this correspondence.

## Conflicts of interest

The authors declare no conflicts of interest.

## References

[1] Tavares C., Memória C.M., Costa L.G.V., Quintão V.C., Antunes A.A., Teodoro D. (2025). Effect of melatonin on postoperative cognitive function in elderly patients submitted to transurethral resection of the prostate under spinal anesthesia. Clinics.

[2] Peden C.J., Miller T.R., Deiner S., Eckenhoff R.G., Fleisher L.A., Jammer I. (2021). Improving perioperative brain health: an expert consensus review of key actions for the perioperative care team. Br J Anaesth.

[3] Hughes C.G., Boncyk C.S., Culley D.J., Fleisher L.A., Leung J.M., McDonagh D.L. (2020). American society for enhanced recovery and perioperative quality initiative joint consensus statement on postoperative delirium prevention. Anesth Analg.

[4] Brodier E.A., Rasmussen L.S. (2021). Postoperative cognitive dysfunction in clinical practice. BJA Educ.

[5] Shin H.W., Kwak J.S., Choi Y.J., Kim J.W., You H.S., Shin H.J. (2024). Efficacy and safety of perioperative melatonin and melatonin agonists for postoperative delirium: a systematic review and meta-analysis. J Int Med Res.

[6] Wang C.M., Zhou L.Y. (2022). Melatonin and melatonergic agents for the prevention of postoperative delirium: a meta-analysis of randomized placebo-controlled trials. Asian J Surg.

[7] Glaser A.P., Mansfield S., Smith A.R., Helfand B.T., Lai H.H., Sarma A.V. (2022). Impact of sleep disturbance, physical function, depression and anxiety on male lower urinary tract symptoms: results from the LURN study. J Urol.

[8] Yin F., He Q.D., Chen J., Gui T.J., Cai R.J., Wang Y. (2023). Benign prostatic hyperplasia associated with white matter hyperintensities in men. Clin Neurol Neurosurg.

[9] Glaser A.P., Cameron A.P., Clemens J.Q., Suskind A.M., Anger J.T., Lai H.H. (2024). Enhanced patient-reported outcome-based approaches for benign prostatic hyperplasia/lower urinary tract symptoms management. BMC Urol.

[10] Tang K., Chen H., Zhang Y., Chen Z., Xu Y., Wang Y. (2023). Efficacy of sleep interventions on postoperative delirium: a systematic review and meta-analysis of randomized controlled trials. Anesth Perioper Sci.

